# Analysis of 16S rRNA Amplicon Sequencing Options on the Roche/454 Next-Generation Titanium Sequencing Platform

**DOI:** 10.1371/journal.pone.0025263

**Published:** 2011-09-23

**Authors:** Hideyuki Tamaki, Chris L. Wright, Xiangzhen Li, Qiaoyan Lin, Chiachi Hwang, Shiping Wang, Jyothi Thimmapuram, Yoichi Kamagata, Wen-Tso Liu

**Affiliations:** 1 Department of Civil and Environmental Engineering, University of Illinois at Urbana-Champaign, Urbana, Illinois, United States of America; 2 Bioproduction Research Institute, National Institute of Advanced Industrial Science and Technology (AIST), Tsukuba, Ibaraki, Japan; 3 W. M. Keck Center for Comparative and Functional Genomics, Roy J. Carver Biotechnology Center, University of Illinois at Urbana-Champaign, Urbana, Illinois, United States of America; 4 Key Laboratory of Adaptation and Evolution of Plateau Biota, Haibei Alpine Meadow Ecosystem Research Station, Northwest Institute of Plateau Biology, Chinese Academy of Sciences, Xining, China; American University in Cairo, Egypt

## Abstract

**Background:**

16S rRNA gene pyrosequencing approach has revolutionized studies in microbial ecology. While primer selection and short read length can affect the resulting microbial community profile, little is known about the influence of pyrosequencing methods on the sequencing throughput and the outcome of microbial community analyses. The aim of this study is to compare differences in output, ease, and cost among three different amplicon pyrosequencing methods for the Roche/454 Titanium platform

**Methodology/Principal Findings:**

The following three pyrosequencing methods for 16S rRNA genes were selected in this study: Method-1 (standard method) is the recommended method for bi-directional sequencing using the LIB-A kit; Method-2 is a new option designed in this study for unidirectional sequencing with the LIB-A kit; and Method-3 uses the LIB-L kit for unidirectional sequencing. In our comparison among these three methods using 10 different environmental samples, Method-2 and Method-3 produced 1.5–1.6 times more useable reads than the standard method (Method-1), after quality-based trimming, and did not compromise the outcome of microbial community analyses. Specifically, Method-3 is the most cost-effective unidirectional amplicon sequencing method as it provided the most reads and required the least effort in consumables management.

**Conclusions:**

Our findings clearly demonstrated that alternative pyrosequencing methods for 16S rRNA genes could drastically affect sequencing output (e.g. number of reads before and after trimming) but have little effect on the outcomes of microbial community analysis. This finding is important for both researchers and sequencing facilities utilizing 16S rRNA gene pyrosequencing for microbial ecological studies.

## Introduction

Next-generation sequencing technology, in particular pyrosequencing using the Roche/454 platform, has been applied to studies in microbial ecology [1]–[3]. Pyrosequencing of 16S rRNA genes (16S pyrotagging or 16S pyrosequencing) has virtually replaced the Sanger-based 16S rRNA sequencing method (e.g., clone library) for microbial diversity analysis because it offers several advantages. For example, thousands of sequences can be obtained by pyrosequencing for a given sample. Additionally, by using barcoded primers to PCR amplify 16S rRNA genes, microbial communities from multiple samples can be simultaneously examined and compared. Pyrosequencing also provides insights into the microbial community structure and diversity at a resolution of 10–100 fold higher and at a cost of 10–100 fold lower than the clone library approach.

Despite its advantages over the traditional approach, there are still some technical issues that need to be addressed in 16S pyrosequencing technology [2], [4]. One of them is the relatively shorter sequence read length, varying from ∼250 bp with the 454 GS FLX platform, 400–500 bp with the 454 GS FLX Titanium platform, to ∼600–800 bp with the upcoming FLX+ 454 platform. Using Illumina and the Life Technologies SOLiD platforms, shorter sequence lengths than those from the 454 platform are generated. Since a sequence length of <500 bp sometimes cannot accurately identify taxonomic affiliation down to a genus or a species level, selection of primers targeting specific variable regions of 16S rRNA gene sequence is critical [3]–[5]. A few candidate primers that target the 16S rRNA genes of domain *Bacteria* have been proposed based on *in*-*silico* analysis and/or experimental results [3], [4], [6]–[8]. Alternative primer sets that target both domains of *Bacteria* and *Archaea* have also been proposed [8].

Likewise, pyrosequencing methods (e.g. the types of chemistry, kit, or fusion primers used) are also equally important. Two types of kits, Roche Titanium LIB-L and Titanium LIB-A, are currently available for sequencing via the 454 Titanium platform. The Roche Titanium LIB-L kit was originally developed for genomic DNA or paired-end DNA sequencing, whereas the Titanium LIB-A kit was designed specifically for bi-directional amplicon sequencing and uses different A and B adapter sequences in the fusion primers. The LIB-A and LIB-L kits differ in the oligonucleotide sequence bound to the capture beads and in the individual primers used in emulsion PCR amplification and downstream enrichment and sequencing. In the LIB-A kit, the sequencing reagent is further divided into two: one portion is used for sequencing from A adaptor (“A” sequencing) and the other for sequencing from B adaptor (“B” sequencing). Alternatively, the LIB-L kit is composed entirely of “A” sequencing reagents. More recently, the LIB-L was suggested for unidirectional sequencing of amplicons (Roche APP No. 001-2009).

Although bidirectional sequencing produces reads that originate from different regions and directions (5′ and 3′ directions), the method complicates downstream analyses and thus the 3′ reads are often discarded from the pyrosequencing output and only the 5′ reads are used in the analyses. Therefore, researchers tend to prefer unidirectional sequencing of 16S amplicons, but it would be wasteful to just use one half of the LIB-A kit. Since the release of the LIB-L kit for unidirectional amplicon sequencing,, no study has yet compared the efficiency between these two kits during project setup or during pyrosequencing, nor were there studies on their differences in microbial community profiles. It is clear that kit selection affects the practical utilization (e.g., cost) of the 454 Titanium platform. Specifically, bi-directional sequencing requires twice the number of barcoded primers compared to unidirectional sequencing. Moreover, if only 5′ reads are used for downstream analyses, the researcher would have to pay twice as much to generate the same number of reads compared to unidirectional sequencing. Alternatively, if the sequencing center utilized the LIB-A kit for unidirectional 16S sequencing, then two kits must be used to have the same amount of reagents for ‘A’ only sequencing. The remaining two portions are discarded, or the sequencing center needs to manage multiple opened kits and find projects looking for ‘B’ only sequencing. Either option raises overall sequencing cost for the customer. Since LIB-L generates only 5′ reads and uses the entire sequencing kit, it appears to be more attractive to use for unidirectional sequencing of 16S amplicons. This study aimed to evaluate the 16S pyrosequencing outputs between the LIB-A and LIB-L kits, and explore their differences, if any, for 16S pyrosequencing on the 454 Titanium platform.

## Results and Discussion

### Pyrosequencing methods

**Figure 1:**
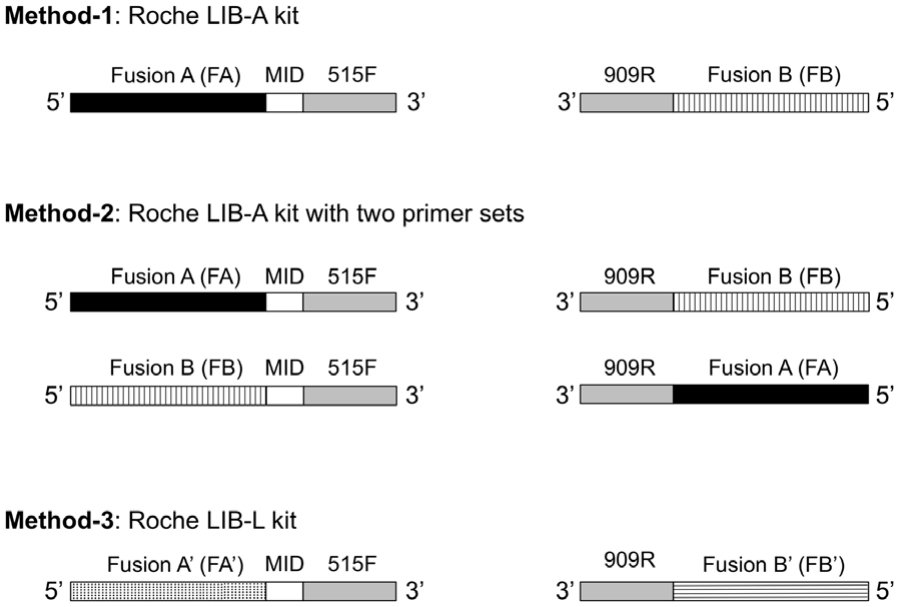
Experimental design showing three methods for 16S pyrotag sequencing. MID stands for multiplex identifier sequence for differentiation of multiplex sequence data sets. Method-1 was performed using LIB-A kit with a primer set of FA-MID-515F and FB-909R. Method-2 was done by LIB-A kit with two primer sets of FA-MID-515F and FB-909R and FB-MID-515F and FA-909R. Method-3 was done using LIB-L kit with a primer set of FA′-MID-515F and FB′-909R.

This study evaluated three different pyrosequencing methods. Method 1 (M1) is the standard method provided by Roche for amplicon pyrosequencing (Fig. 1). It uses the Titanium LIB-A kit for bi-directional sequencing (ROCHE TCB-09013) with forward and reverse primers barcoded with “A” and “B” adaptor sequences, respectively. The 5′ “A” adaptor of the forward primer “FA”(CGTATCGCCTCCCTCGCGCCATCAG)-MIDs-515F [8] is barcoded since only “A” direction 16S pyrosequencing reads are kept and used for the downstream microbial community analyses. The reverse primer, “FB”(CTATGCGCCTTGCCAGCCCGCTCAG)-909R [8], is not barcoded and reads from “FB” are discarded.

Method 2 (M2) is a new option for 16S pyrosequencing designed in this study. This method also uses the LIB-A kit but utilizes two primer pairs: FA-MIDs-515F and FB-909R, and FB-MIDs-515F and FA-909R (Fig. 1). The first primer set is identical to the primer set for Method-1, while in the second primer set the “A” and “B” adaptors are switched onto the reverse and forward primers, respectively. With this configuration, the “A” adaptor remains barcoded for the first primer set as in Method 1, whereas in the second primer set the “B” adaptor is barcoded in the forward primer. The A and B pools are kept separate through emulsion PCR (emPCR) and mixed evenly just before sequencing. This method generates 5′-only sequence data and allows the full use of the entire Titanium LIB-A sequencing kit.

Method 3 (M3) is the second option provided by Roche for amplicon pyrosequencing (ROCHE APP No. 001-2009). It uses the Titanium LIB-L kit that was previously used only for 5′-only sequencing of paired-end DNA, cDNA, genomic DNA, or metagenomic DNA via the 454 GS FLX Titanium platform. In this study, Method-3 is carried out with the standard Roche Titanium A and B adaptors (FA′ and FB′ in Fig. 1) rather than the Titanium amplicon adaptors (FA and FB in Fig. 1). Further, only one primer set, FA′(CCATCTCATCCCTGCGTGTCTCCGACTCAG)-MIDs-515F and FB′(CCTATCCCCTGTGTGCCTTGGCAGTCTCAG)-909R (Fig. 1), was used for this method to generate 5′-only reads while also utilizing the entire LIB-L kit.

### Comparison of pyrosequencing methods for microbial community analysis

We first analyzed the results of the three pyrosequencing methods performed on 10 environmental samples based on the total numbers of sequences and read quality obtained (Table 1). Among them, M3 gave the highest number of reads before and after sequence quality trimming, but with slightly lower read quality of sequences (67.3% of good quality reads after trimming). M2 and M3 produced 1.5 and 1.6 fold more sequence reads after trimming than M1 (Table 1). Total numbers of reads before and after trimming varied among those 10 samples (Table S1), and most samples (8 out of 10) had more sequence reads using M2 and M3 than M1 before and after quality trimming.

**Table 1:** Total number of sequences obtained via three pyrosequencing methods using one lane of 16^th^ region.

Method*	Before trimming	After trimming		
	No. of reads	No. of reads	% of reads	Length [nt]
Method-1 (n = 2)	13730±996	11835±960	85.7±0.8	377±0
Method-2 (n = 2)	21469±1865	18124±1457	84.8±0.6	377±0
Method-3 (n = 2)	28713±25	19153±1776	67.3±5.9	377±0

All data are given as an average value of technical replicates (duplicates (n = 2)). The values show the variation obtained from the duplicates.

*Method-1, unidirectional method using Roche Titanium LIB-A kit; Method-2, unidirectional sequencing method using Roche Titanium LIB-A kit with dual primer sets (515F-FA-MIDs & 909R-FB and 515F-FB-MIDs and 909R-FA); Method-3, unidirectional sequencing method using Roche Titanium LIB-L kit.

**Figure 2:**
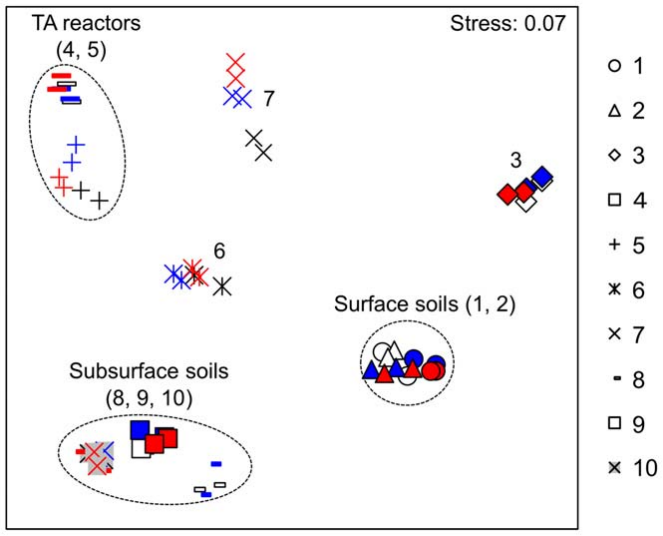
Multidimensional scaling plot (MDS) obtained by 454 pyrosequencing with three methods, Method-1 (M1), Method-2 (M2), and Method-3 (M3), for 10 environmental samples (1, surface soil-1; 2, surface soil-2; 3, Drinking water biofilm; 4, TA-bioreactor (TAJun06); 5, TA-bioreactor (TAAug09); 6, Primary anaerobic digester; 7, Groundwater; 8, Peat soil; 9, Glacial deposit soil-1; 10, Glacial deposit soil-2). Methods-1, -2, and -3, are shown in black and white, blue, and red, respectively.

**Figure 3:**
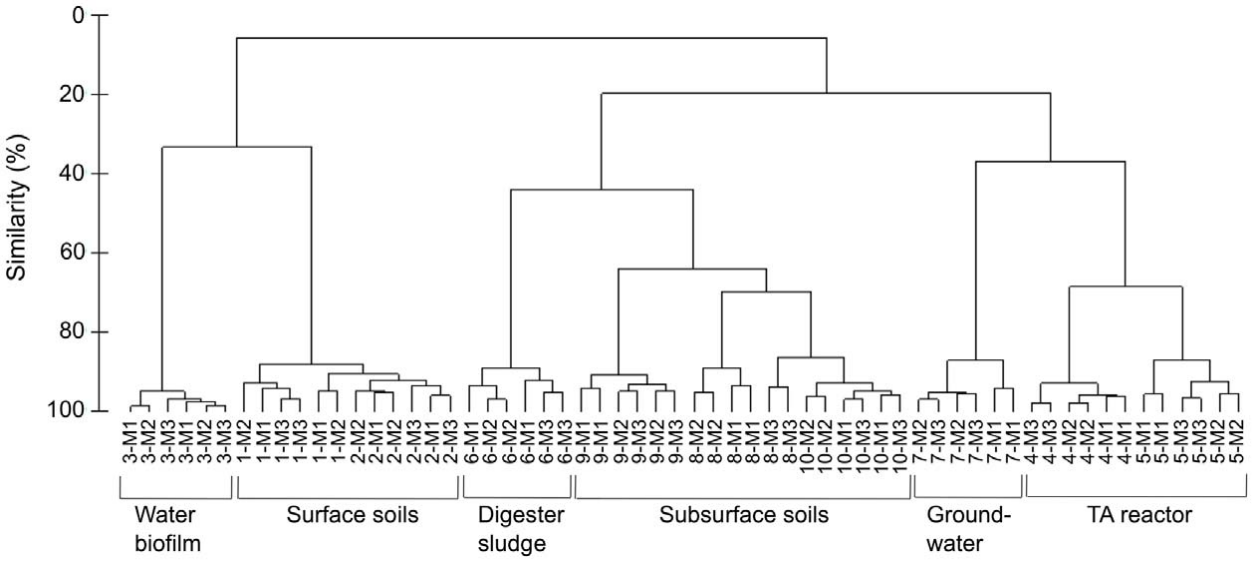
Similarity tree using Bray-Curtis metrics obtained by 454 pyrosequencing with three methods, Method-1 (M1), Method-2 (M2), and Method-3 (M3), for 10 environmental samples (1, surface soil-1; 2, surface soil-2; 3, Drinking water biofilm; 4, TA-bioreactor (TAJun06); 5, TA-bioreactor (TAAug09); 6, Primary anaerobic digester; 7, Groundwater; 8, Peat soil; 9, Glacial deposit soil-1; 10, Glacial deposit soil-2).

The similarity in microbial compositions among those ten samples was further examined and compared. Multidimensional scaling plot (MDS) analysis showed that samples from the same environment closely clustered together irrespective of the sequencing methods used (Fig. 2). Samples from anaerobic environments (terephthalate (TA)-degrading methanogenic reactor, groundwater, subsurface soils, and primary digester sludge) appeared to be more similar to each other than those from aerobic environments (drinking water biofilms and surface soils). The similarity tree (Fig. 3) based on Bray-Curtis algorithm also clearly showed the same clustering pattern observed in MDS analysis. Mantel test was further applied to compare the microbial composition for a given sample obtained by those three sequencing methods. Paired-wise comparison of the similarity distance matrix suggested no significant difference in microbial composition obtained from any two different methods: M1 vs M2 (R>0.98, P = 0.001), M1 vs M3 (R>0.95, P = 0.001), and M2 vs M3 (R>0.95, P = 0.001).

The microbial community diversity indices for 10 environmental samples obtained from the three pyrosequencing methods were further compared (Table S1). All methods gave similar numbers of OTUs, Shannon-Weaver index, and Chao 1 in all samples except for the peat soil which gave two to three times higher numbers of OTUs and Chao 1 using M2 than M1 and M3. The percentages of bacterial and archaeal sequences obtained from those three methods were also similar in all samples (Table S1).

**Figure 4:**
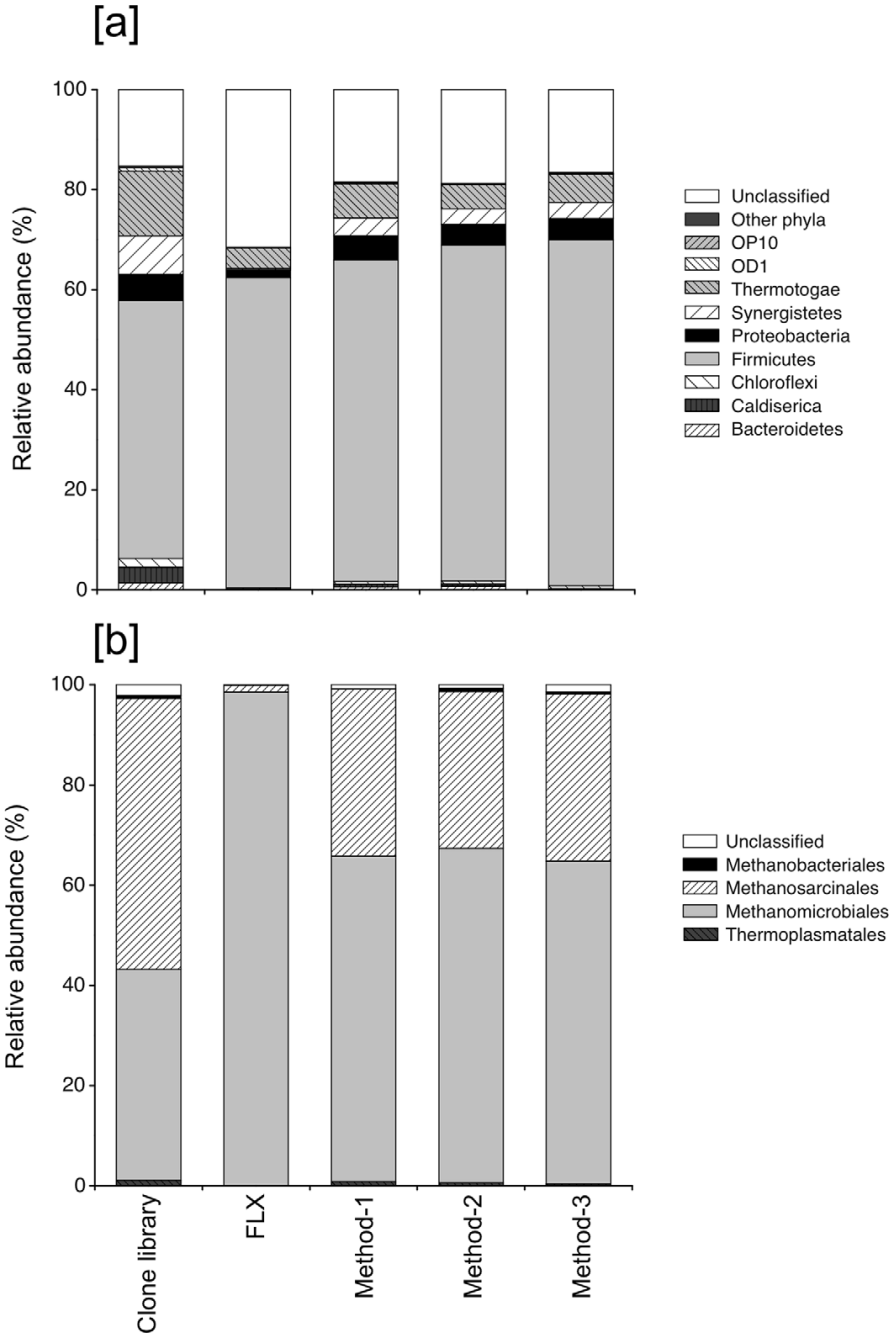
Effect of pyrosequencing methods on microbial compositions in TAJun06 (June 2006 sample of anaerobic TA-degrading bioreactor). [a] Relative abundance of bacterial phyla and [b] relative abundance of archaeal orders. The data of clone library was derived from our previous study [9]. FLX data were obtained from 16S pyrosequencing using a FLX platform (unpublished data). A bacterial primer set (27F and 534R) and an archaeal primer set (A1F and 571R) were used for the FLX pyrosequecing.

Finally, microbial community compositions obtained from those three pyrosequencing methods by using the TA-degrading bioreactor sample (TAJun06) as a reference were compared, since the bacterial and archaeal community compositions in this sample have been extensively studied with nearly full-length 16S rRNA gene sequences clone library (287 and 359 clones for the *Bacteria* and *Archaea*, respectively) [9]. The microbial community compositions of domains *Bacteria* and *Archaea* obtained using M1, M2 and M3 were very similar to each other (Figs. 4a and 4b). Community compositions from those three methods in general gave similar results with those obtained by the conventional clone library procedure, where higher relative abundance of *Firmicutes* and unclassified bacteria in the domain *Bacteria*, and *Methanomicrobiales* in the *Archaea*, and lower relative abundance of *Thermotogae* in the *Bacteria* and the *Methanosarcinales* in the *Archaea* were observed (Figs. 4a and 4b). However, the differences in both bacterial and archaeal community compositions between the three methods and the clone library were much smaller than those between FLX data (obtained using 454 GS FLX platform (unpublished data)) and the clone library (Figs. 4a and 4b). In the FLX data, percentages of unclassified sequences in the *Bacteria* were much higher than others (Fig. 4a) due to the shorter read length (approx. 230–250 bp) [5]. The shorter read length further led to incorrect assignment of archaeal community compositions with overrepresentation of the *Methanomicrobiales* (Fig. 4b).

Liu et al. [4] reported that no marked differences between 250 bp (e.g. FLX platform) and 400 bp (e.g. Titanium platform) of read length were observed in taxonomic identification by their *in-silico* analyses. In contrast, our experimental results indicated that all three methods on the Titanium platform allowed for more accurate analysis of microbial communities compared to the shorter reads obtained from the FLX platform, and produced results similar to that obtained using conventional Sanger sequencing. Nevertheless, it should be noted that the outcomes of all these sequencing methods are likely to be affected by biases associated with the DNA extraction step, primer selection and design, target variable regions of 16S rRNA genes, and PCR amplification steps [3], [4], [6]–[8], [10]–[14]. In addition, the clone library method with Sanger sequencing has its own possible biases in cloning steps (e.g. DNA ligation and transformation procedures) [15]. In particular, the difference in the primers used for clone library (27F-1391R for bacteria and 4aF-1391R for archaea), the FLX platform (27F-534R for bacteria and A1F-571R for archaea), and the Titanium platform (515F-909R for bacteria and archaea) may also affect the results of microbial community compositions obtained. To date, domain specific primers (bacterial or archaeal primers) have been widely used for microbial community analysis because of higher coverage than universal primers targeting both bacteria and archaea. This might have led to slight differences in the microbial community compositions obtained from the three pyrosequencing methods M1–M3 and the clone library. However, it should be noted that there are no primers sets that can target all bacterial 16S rRNA gene sequences, all archaeal 16S rRNA gene sequences, or both [8].

### Conclusions

Our results clearly demonstrated that different pyrosequencing methods could drastically affect sequencing output (e.g. number of reads before and after trimming), but did not alter the outcome of the microbial community analysis. M1 is not recommended if single end-reads are needed due to its higher cost and/or kit waste and management. Yet, it remains the only viable option for bi-directional sequencing. M2 produced an equal number of quality reads as M1 with unidirectional sequencing and allowed the full use of the LIB-A emPCR kit. However, it was the least desirable method as it was more labor intensive and had a higher consumable cost than the other two methods due to the fact that two different PCR reactions and two different primer sets were required for each sample. With virtually similar results in microbial community analyses between the three methods, M3 was clearly the optimal method for unidirectional sequencing of 16S rRNA gene amplicons since it provided the highest number of reads with the same cost as M1 and allowed the full use of emPCR kit LIB-L.

## Materials and Methods

### Environmental samples and DNA extraction

Environmental samples used in this study were two alpine meadow surface soil samples from Qinghai-Tibetan plateau, China, one residential area drinking water biofilm in Urbana, Illinois, USA, two samples from a TA-degrading bioreactor (TAJun06 and TAAug09) [9], one sludge sample collected from a primary anaerobic digester at Urbana, Illinois, USA, one groundwater sample collected in Illinois, USA, one peat soil collected in Illinois, USA, and two glacial deposit soil samples collected in Illinois, USA. Genomic DNAs were extracted using a protocol described previously [16] or the FastDNA SPIN kit for soil (MP biomedicals, USA) according to the manufacturer's instruction.

### PCR amplification and 454 pyrosequencing

The 16S rRNA gene was PCR-amplified with the primer pairs described above (details provided in Table S1) using Bullseye standard Taq DNA polymerase 2.0× master mix (MIDSCI, St. Louis, MO, USA). The PCR for each method was carried out in 50 µl reaction volumes in S1000 Thermal Cycler (BioRad, Hercules, CA, USA) with the following parameters: initial denaturation at 94°C for 3 min, followed by 30 cycles of 94°C for 40 s, 56°C for 1 min, and 72°C for 1 min with a final extension at 72°C for 10 min. PCR products were run on 1.5% agarose gel electrophoresis and the DNA band with the correct size was excised and purified using Wizard® SV Gel and PCR Clean-Up System (Promega, St. Louis, MO, USA). Equal amount of purified PCR products were pooled for subsequent 454 pyrosequencing.

454 pyrosequencing was carried out on the Titanium platform (Roche/454 Life Sciences) at the W.M. Keck Center, part of the Roy J. Carver Biotechnology Center at the University of Illinois at Urbana-Champaign. The barcoded and pooled amplicons were checked on an Agilent Bioanalzyer DNA7500 chip for the absence of primer-dimers, quantitated with Qubit assays (Invitrogen), and diluted to 1×10^8^ molecules/ul. Emulsion PCR was set up according to Roche's protocols for the three methods, each in duplicate. Sequencing was performed using 16-region gaskets and each sample was run in two lanes. Sequencing results were analyzed with Roche software version 2.5.3, signal processing for amplicons.

### Data Analysis

The pyrotags were sorted by barcodes (MIDs) to set up 10 libraries for protocol comparison. All sequences were trimmed based on the initial data processing in RDP pyrosequencing pipeline [17] with default parameters (max number of N's = 0 and minimum average quality score = 20) using forward and reverse primer sequences. Chimera sequences were detected and removed by Bellerophon. The trimmed sequences were aligned in RDP aligner and clustered using the Complete Linkage Clustering tool in the RDP. Based on the clustered sequence data, microbial community diversity indices (number of OTUs, Shannon-Weaver (H′) index, Chao 1) were obtained using a cutoff value of 97% sequence similarity. RDP classifier was used for taxonomic assignments of the 16S pyrosequencing reads at 70% confidence level [17]. A similarity tree of 10 environmental samples was constructed from Bray-Curtis distance metrics based on microbial community compositions. To test the significant similarity between any two pyrosequencing methods (M1, M2, and M3), a Mantel test was conducted. Multidimensional scaling plot (MDS) analysis was performed using Primer 6 (http://www.primer-e.com/) based on Bray-Curtis distance matrices obtained from microbial community compositions (the relative abundance of archaeal and bacterial phyla).

## Supporting Information

Table S1 **Sequences obtained via pyrosequencing of 10 environmental samples.**
(PDF)
