# Supplementary material for: Analysis of 16S rRNA Amplicon Sequencing Options on the Roche/454 Next-Generation Titanium Sequencing Platform
Source: PLoS One. 2011 Sep 23;6(9):e25263. doi: 10.1371/journal.pone.0025263 (PMC3179495; doi:10.1371/journal.pone.0025263)
Supplement: Table S1 — Sequences obtained via pyrosequencing of 10 environmental samples. (PDF) [file pone.0025263.s001.pdf]

Supplementary Table S1. Sequences obtained via pyrosequencing of 10 environmental samples

No.	Sample	Method*	Before trimming	After trimming			Diversity index**			Classification of reads at domain level		
										Percentage [%]		
			No. of reads	No. of reads	% of reads	Length [nt]	No. of OTUs	H'	Chao	Bacteria	Archaea	Unclassified
1	Tibet soil-1	Method-1 (n=2)	1242 ± 110	1093 ± 112	87.9 ± 1.2	376 ± 0	650 ± 74	6.2 ± 0.1	1958 ± 164	98.4 ± 0.4	1.7 ± 0.4	0.0 ± 0.0
		Method-2 (n=2)	2290 ± 559	1988 ± 468	87.0 ± 0.8	376 ± 0	882 ± 147	6.3 ± 0.1	1888 ± 327	98.8 ± 0.3	1.2 ± 0.3	0.0 ± 0.0
		Method-3 (n=2)	3387 ± 601	2139 ± 56	64.9 ± 9.9	376 ± 0	837 ± 31	6.2 ± 0.1	1670 ± 81	99.6 ± 0.1	0.4 ± 0.1	0.0 ± 0.0
2	Tibet soil-2	Method-1 (n=2)	1808 ± 162	1596 ± 135	88.3 ± 0.5	376 ± 0	762 ± 46	6.3 ± 0.0	1565 ± 114	98.6 ± 0.2	1.4 ± 0.1	0.1 ± 0.1
		Method-2 (n=2)	2511 ± 248	2199 ± 222	87.6 ± 0.2	376 ± 0	1002 ± 109	6.5 ± 0.0	2144 ± 108	98.3 ± 0.0	1.8 ± 0.1	0.1 ± 0.1
		Method-3 (n=2)	2720 ± 195	1762 ± 97	65.4 ± 8.2	376 ± 0	848 ± 50	6.3 ± 0.1	1968 ± 250	99.3 ± 0.2	0.7 ±0.2	0.0 ± 0.0
3	Drinking water biofilm	Method-1 (n=2)	1883 ± 176	1663 ± 178	88.2 ± 1.2	375 ± 0	316 ± 26	4.6 ± 0.0	610 ± 30	100.0 ± 0.0	0.0 ± 0.0	0.0 ± 0.0
		Method-2 (n=2)	1575 ± 125	1389 ± 110	88.2 ± 0.0	375 ± 0	279 ± 10	4.4 ± 0.0	538 ± 109	99.9 ± 0.0	0.1 ± 0.1	0.0 ± 0.0
		Method-3 (n=2)	2084 ± 46	1406 ± 143	67.6 ± 8.3	375 ± 0	296 ± 46	4.5 ± 0.1	568 ± 71	100.0 ± 0.0	0.0 ± 0.0	0.0 ± 0.0
4	TA-bioreactor (Jun06)	Method-1 (n=2)	1186 ± 68	1050 ± 65	88.5 ± 0.4	377 ± 0	95 ± 11	2.9 ± 0.1	237 ± 98	78.3 ± 0.5	21.8 ± 0.6	0.0 ± 0.0
		Method-2 (n=2)	1695 ± 211	1497 ± 189	88.3 ± 0.2	377 ± 0	130 ± 10	3.0 ± 0.0	270 ± 30	78.8 ± 0.2	21.2 ± 0.2	0.0 ± 0.0
		Method-3 (n=2)	1886 ± 67	1390 ± 18	73.8 ± 3.5	377 ± 0	110 ± 7	2.8 ± 0.0	216 ± 43	80.9 ± 0.2	19.1 ± 0.2	0.0 ± 0.0
5	TA-bioreactor (Aug10)	Method-1 (n=2)	1308 ± 69	1155 ± 70	88.2 ± 0.7	377 ± 0	115 ± 11	3.2 ± 0.1	204 ± 1	68.8 ± 0.5	31.3 ± 0.4	0.0 ± 0.0
		Method-2 (n=2)	1499 ± 107	1319 ± 85	88.0 ± 0.6	378 ± 0	110 ± 6	2.8 ± 0.0	222 ± 41	58.5 ± 1.5	41.5 ± 1.5	0.1 ± 0.1
		Method-3 (n=2)	2171 ± 28	1540 ± 140	70.8 ± 5.5	378 ± 0	143 ± 18	2.9 ± 0.0	303 ± 100	61.3 ± 1.4	38.7 ± 1.4	0.1 ± 0.1
6	Primary anaerobic digester	Method-1 (n=2)	926 ± 71	643 ± 56	69.3 ± 0.7	375 ± 0	230 ± 8	4.7 ± 0.0	509 ± 8	96.3 ± 0.7	3.7 ± 0.7	0.1 ± 0.1
		Method-2 (n=2)	2084 ± 154	1351 ± 103	64.8 ± 0.1	376 ± 0	373 ± 25	4.8 ± 0.1	863 ± 64	94.3 ± 0.7	5.4 ± 0.4	0.2 ± 0.1
		Method-3 (n=2)	2737 ± 201	1386 ± 252	50.2 ± 5.5	376 ± 0	350 ± 53	4.6 ± 0.1	760 ± 120	95.9 ± 0.6	4.0 ± 0.5	0.1 ± 0.1
7	Groundwater	Method-1 (n=2)	559 ± 78	488 ± 75	87.2 ± 1.3	378 ± 0	85 ± 11	2.8 ± 0.1	217 ± 39	45.4 ± 0.8	54.3 ± 1.0	0.4 ± 0.2
		Method-2 (n=2)	1536 ± 116	1308 ± 93	85.2 ± 0.4	379 ± 0	125 ± 7	2.7 ± 0.0	255 ± 11	37.6 ± 0.6	62.0 ± 0.8	0.5 ± 0.2
		Method-3 (n=2)	2290 ± 100	1718 ± 139	74.9 ± 2.8	379 ± 0	144 ± 14	2.7 ± 0.0	323 ± 5	34.7 ± 0.9	65.0 ± 0.9	0.4 ± 0.1
8	Peat soil	Method-1 (n=2)	649 ± 32	544 ± 24	83.8 ± 0.4	378 ± 0	283 ± 9	5.2 ± 0.0	746 ± 48	67.3 ± 1.5	32.1 ± 1.5	0.7 ± 0.0
		Method-2 (n=2)	2104 ± 124	1752 ± 66	83.4 ± 1.8	378 ± 0	693 ± 27	5.8 ± 0.0	2129 ± 36	71.0 ± 0.1	28.2 ± 0.3	0.4 ± 0.4
		Method-3 (n=2)	2355 ± 201	1521 ± 267	64.1 ± 5.9	379 ± 0	285 ± 24	4.0 ± 0.0	642 ± 21	72.1 ± 0.8	26.9 ± 0.9	1.1 ± 0.1
9	Glacial deposit soil-1	Method-1 (n=2)	1362 ± 86	1161 ± 88	85.2 ± 1.1	379 ± 1	359 ± 20	5.1 ± 0.1	743 ± 13	87.7 ± 1.5	9.7 ± 1.6	2.7 ± 0.0
		Method-2 (n=2)	1337 ± 19	1150 ± 13	86.0 ± 0.3	378 ± 0	404 ± 19	5.3 ± 0.1	936 ± 4	90.0 ± 0.8	7.7 ± 0.0	2.3 ± 0.4
		Method-3 (n=2)	2265 ± 126	1422 ± 210	62.4 ± 5.8	378 ± 0	367 ± 18	4.7 ± 0.6	777 ± 81	86.6 ± 0.9	9.3 ± 0.5	4.1 ± 0.8
10	Glacial deposit soil-2	Method-1 (n=2)	1361 ± 88	1164 ± 97	85.4 ± 1.6	378 ± 0	252 ± 25	4.2 ± 0.1	612 ± 46	76.2 ± 0.9	22.3 ± 0.9	1.5 ± 0.0
		Method-2 (n=2)	1563 ± 4	1301 ± 39	83.2 ± 2.7	378 ± 0	321 ± 14	4.6 ± 0.0	889 ± 116	78.9 ± 0.1	19.7 ± 0.4	1.4 ± 0.3
		Method-3 (n=2)	2116 ± 70	1365 ± 109	64.8 ± 7.3	378 ± 0	279 ± 14	4.2 ± 0.0	751 ± 27	75.2 ± 1.4	23.6 ± 1.3	1.3 ± 0.1

All data are given as an average value of technical replicates (duplicates (n=2)). The values show the variation obtained from the duplicates.

*Method-1, unidirectional method using Roche Titanium LIB-A kit; Method-2, unidirectional sequencing method using Roche Titanium LIB-A kit with dual primer sets (515F-FA-MIDs & 909R-FB and 515F-FB-MIDs and 909R-FA); Method-3, unidirectional sequencing method using Roche Titanium LIB-L kit

**Diversity indexes were based on a taxonomic cutoff of 97%
